# Affective Risk Associated With Menstrual Cycle Symptom Change

**DOI:** 10.3389/fgwh.2022.896924

**Published:** 2022-07-22

**Authors:** Jeff Kiesner, Tory A. Eisenlohr-Moul, Giulio Vidotto

**Affiliations:** ^1^Department of Developmental and Social Psychology, University of Padova, Padua, Italy; ^2^Department of Psychiatry, University of Illinois at Chicago, Chicago, IL, United States; ^3^Department of General Psychology, University of Padova, Padua, Italy

**Keywords:** affective disorders, risk, menstrual cycle, depression, anxiety, women

## Abstract

In the present study we test whether cyclical changes in affective symptoms of the menstrual cycle are associated with higher mean levels of those same symptoms. Using prospective daily reports across two full menstrual cycles, from two samples of female University students (*n* = 213; *n* = 163), we applied both quartic polynomial regressions and cosine regressions to model cyclical change in symptoms, and to test for mean-level differences in symptoms across the resulting trajectory patterns. Counter to prior findings, but consistent with theoretical expectations, these results show that females who experience menstrual cycle-related changes in affect (whether a perimenstrual or mid-cycle increase) are at risk for higher average levels of affective symptoms. These results suggest that the mid-cycle group should be recognized as a target for future research that is associated with increased risk for chronic negative affective symptoms.

## Introduction

Decades of research have provided a wealth of information regarding the presence of psychological and physical changes associated with the menstrual cycle. Of central importance have been insights to steroidal triggers (1–3); genetic risks (2, 4, 5), and a deeper understanding of the associations between diverse symptom types (6, 7). Given these advances, it is surprising that efforts to understand the role of these symptoms in a broader context of clinical psychology have been extremely limited. Indeed, most studies on sex or gender differences in affective disorders do not measure or control for menstrual cycle-related symptoms, or even the specific timing in the menstrual cycle in which measurement was conducted, which could easily bias estimates of levels and prevalence rates of depression among females (8). One very simple but potentially important question has been almost completely ignored: *Is there an association between the degree of affective change across the menstrual cycle and the average level of affective symptoms experienced?* Answering this question could provide insight regarding risk for affective disorders associated with menstrual cycle-related symptoms beyond the immediate effects of the menstrual cycle.

One study that did test for the association between menstrual cycle-related changes in depression/anxiety and mean levels of those same symptoms found that participants with a strong perimenstrual increase in symptoms (termed a premenstrual syndrome pattern [PMS pattern] of change) showed the lowest mean levels of symptoms, whereas those with no cyclic change in symptoms showed the highest mean levels of symptoms (9). However, three issues necessitated caution in interpreting and generalizing those findings. First, a lack of theoretical foundation or explanation for the observed difference indicates the need for further theoretical and empirical work. Second, the analytic method used by Kiesner (9) for creating trajectory groups was novel, and previously untested, thus requiring replication, and possible comparison with other methods. Third, the mere fact that no previous studies had attempted to directly test this association requires that replication be demonstrated.

It should be noted that two other studies have provided *indirect* support for the idea that females who experience premenstrual symptoms may benefit from lower average levels of the same symptoms. First, Metcalf and Livesey (10) found that participants who identified themselves as having PMS demonstrated a clustering of positive mood days around ovulation, whereas those not reporting PMS did not demonstrate this clustering of positive mood days. This would suggest that among those with PMS, there is both a premenstrual worsening of negative affect *and* a mid-cycle improvement in mood, which may compensate and provide an overall benefit in mood for those with PMS. In a second study, normal controls, PMDD patients, and patients with brief recurrent depression, were compared on a measure of sadness, and although no significant differences were observed in mean levels, the normal controls showed a higher mean level of sadness as compared to PMDD patients, the lack of significance may have been attributable to small sample sizes [*n*'s = 8, 15, and 9, respectively; (11)].

Although these past studies provide some evidence that females who experience PMDD/PMS symptoms may have *lower mean levels* of negative affect, at least three theories could be used to predict the exact opposite. First, Kiesner (12) proposed that, among adolescent girls, recurrent physical and psychological symptoms of the menstrual cycle may lead to compromised engagement in important developmental activities, which may then lead to problems of adjustment such as depressogenic attributional biases, and increased body-image dissatisfaction, which may further lead to increased risk for affective symptoms and disorders (Menstrual Cycle-Response and Developmental Affective-Risk model; MCR-DAR model). Second, the *kindling hypothesis* of depression (13, 14), postulates that initial episodes of depression depend on concurrent psychosocial stressors, but that later episodes are driven by permanent neurophysiological changes that are sequelae of the earlier episodes. To the extent that PMS/PMDD symptoms may result in recurring psychophysiological changes associated with depression, similar *lasting* changes may also ensue, resulting in higher mean levels of depression across time. Third, (15) argued that recurrent negative stressors represent *repeated hits* to allostasis which results in *wear and tear* to important physiological systems involved in stress response (e.g., HPA axis). Lasting changes to these stress adaptation systems are predicted to result in both physical illness (e.g., cardiovascular, immune dysregulation) and psychological symptoms and illness such as depression (16). Because recurrent menstrual cycle-related symptoms may be experienced as repeated hits to allostasis, this model could also be used to predict long-term negative outcomes associated with recurrent menstrual cycle-related symptoms. Thus, all three of these *sensitization hypotheses* predict that cyclically recurring negative symptoms may result in risk for higher mean-levels of depressive symptoms and pathology.

Consistent with the idea that recurrent menstrual cycle-related symptoms may be associated with increased risk for the development of affective disorders is research showing that concurrent associations exist between PMS/PMDD and other affective disorders (17–21), and longitudinal studies showing that baseline PMS/PMDD symptoms predict later affective disorders (22–24). However, none of these studies specifically tested for associations between cyclical change and mean levels, and only one used daily reports of symptoms for multiple cycles (23), and that study is limited by a small sample size (*n* = 8 PMDD; and *n* = 9 without PMDD). Thus, conclusions are very limited.

A final issue that must be considered is the recent discovery that cyclical changes associated with the menstrual cycle are not limited to a *perimenstrual* increase in symptoms, but may also involve a mid-cycle increase in symptoms (symptoms peaking roughly halfway between the onset of two different menses). Specifically, whereas some females experience a perimenstrual increase in symptoms and a mid-cycle improvement, others experience a mid-cycle increase in symptoms, and a perimenstrual improvement. These mid-cycle increases have been observed for a variety of symptoms, including negative affect (9), sleep difficulties (25), headaches (26), and a variety of other physical and psychological symptoms, including attributional bias (6). If the *sensitization hypotheses* described above are true, then we should expect higher mean levels of affective symptoms for females who experience either perimenstrual increases in symptoms (e.g., PMS/PMDD), or mid-cycle increases in symptoms.

It should be noted that the goal of the present study is not to challenge the idea that PMDD patients experience a significant decrease in symptoms following menstrual onset–this is part of the diagnostic criteria–but rather, whether the cyclical changes will put them at risk for experiencing higher average levels of negative affect across time (collapsing across menstrual cycle phase).

In the present study, we test whether an individual's pattern of change in affective symptoms across the menstrual cycle (perimenstrual increase, mid-cycle increase, no cyclical change) is associated with the individual's mean level of those same affective symptoms. To test for these associations, two separate samples are analyzed using two different analytic methods.

## Methods

### Participants

Two different samples drawn from the same university are included in the present report. It should be noted that data from both samples have previously been published, and therefore the following *methodological* description overlaps significantly with information presented elsewhere [e.g., (6, 9)].

For both samples, first-year female psychology students were asked to participate, and efforts were made to include females both with and without menstrual difficulties (see next paragraph). Individuals could not participate if they were using hormonal contraceptives or therapy. Individuals who had been diagnosed with a psychological or medical condition for which they had been, or were being treated, were welcomed to participate. That is to say, pre-existing illness (physical or psychological) was not used as an exclusionary criterion. However, participants with a seasonal illness (cold/flu) were asked to wait until it passed before starting (e.g., waiting till the onset of a subsequent menstruation). Participation was anonymous, voluntary, and did not result in compensation. The Ethics Committee of Psychological Research, of the University of Padova, approved this study, and all participants signed an informed consent.

Recruitment for both samples was conducted at the end of lectures in first-year psychology classes after all male students were asked to leave the lecture hall, and a brief explanation of the study was given without providing specific information regarding study hypotheses. A central point that was emphasized during the explanation was the importance of including females who have very different experiences during the menstrual cycle, and that it would be equally as important for females with and without menstrual difficulties to participate. For example, it was specifically stated that for the success of the study it was equally as important to have females who experience menstrual cycle-related changes (physical or psychological) and those who experience no changes whatsoever. This was emphasized to reduce self-selection bias that could lead to more individuals with significant cyclical changes choosing to participate. Other points that were emphasized included (1) the personal nature of the questions, and (2) the degree of participation required (daily questionnaires for two menstrual cycles; ~60 days). These points were emphasized to avoid surprise on the part of participants. The overall presentation, including questions and responses, lasted approximately 15 min.

Participants from sample 1 were 213 female university students with a mean age of *M* = 21.29 years (*SD* = 4.01; data collected during the years 2007~2008). The average length of the two menstrual-cycles were *M* = 29.57 days for cycle 1, and *M* = 30.48 days for cycle 2 (average length of two consecutive cycles *M* = 60.05 days). The average number of questionnaires for each participant was *M* = 55.09. Thus, on average, participants missed only 5 of the daily questionnaires across the two menstrual cycles, and a total of 11,735 questionnaires were included in the following analyses.

Participants from sample 2 were 163 female university students with a mean age of *M* = 19.54 years (*SD* = 1.22; data collected during the years 2013-2014). The average length of the two menstrual-cycles was *M* = 29.78 days for cycle 1, and *M* = 30.32 days for cycle 2 (average length of two consecutive cycles *M* = 60.09 days). The average number of questionnaires for each participant was *M* = 57.26. Thus, on average, participants missed approximately 3 of the daily questionnaires across the two menstrual cycles, and a total of 9,334 questionnaires were included in the following analyses.

Table 1 provides descriptive and summary information for the two samples. Of note, is the low level of any psychiatric diagnoses, and especially the complete absence of any PMS/PMDD diagnoses. We believe this is due to the relatively young age of the sample and the fact that menstrual cycle-related diagnoses are generally not recognized or treated in Italy.

**Table 1:** Descriptive and demographic characteristics of both samples.

**Variable**	**Sample 1**	**Sample 2**
	**Mean (S.D.) *n* (%)**	**Mean (S.D.) *n* (%)**
Age	21.29 (4.01)	19.54 (1.22)
Age at menarche	12.30 (1.45)	11.96 (1.29)
Children	2 (1%)	1 (<1%)
Smoker	48 (23%)	37 (23%)
Illness	18 (8.5%)	13 (8%)
**Illnesses named**		
	Allergy (1)	Allergy (2)
	Anorexia (1)	Asma (2)
	Anxiety (2)	Celiac (1)
	Asma (1)	Deep vein
	Cardiac (1)	Thrombosis (1)
	Depression (1)	Depression (1)
	Epilepsy (2)	Eosinophilic
	Herniated disk (1)	Esophagitis (1)
	HPV (1)	Epilepsy (1)
	Hypertension (1)	PCOS (1)
	Migraines (1)	Spotting (1)
	PCOS (1)	Thyroid (2)
	Stargardt (1)	
	Tachycardia (1)	
	Thyroid (1)	
	Tooth extraction (1)	

### Measures

#### Online Questionnaire and Procedure

With the use of an individual password, participants had access to an online questionnaire. All questions referred to the last 24 h. Participants were asked to begin completing questionnaires on the first or second day of menstruation, and to indicate on which day they were starting. Because data collection was conducted using an on-line questionnaire, the time and date of completion was automatically recorded and saved with each questionnaire. Participants were asked to complete one questionnaire each day (at approximately the same time each day) and were prompted to respond to all questions if any responses were skipped (thus, there are no missing responses within any questionnaires). However, if they were not able to do so, or accidentally missed a day, the online questionnaire also allowed participants to complete one questionnaire for the prior day, and one questionnaire for the actual day. To control for this, the first question on each questionnaire was whether it was in relation to “yesterday” or “today.” Participants were asked not to go back more than 1 day (“yesterday”).

#### Depression/Anxiety

The depression/anxiety measure was based on two subscales: “*Depression*” (4 items; *in the last 24 h did you feel down?; in the last 24 h did you feel depressed?; in the last 24 h did you feel sad?; in the last 24 h did you have crying spells?*), and “*Anxiety*” (2 items: *in the last 24 h did you feel anxious?*; *in the last 24 h did you feel tense or nervous?*). For sample 1, responses were given on a 5-point response scale ranging from “*Not at all*” to “*Very much*,” using a table format so that each response fell into a discreet column; whereas for sample 2, each question was presented individually with a sliding scale that used only two anchors “*Not at All*” and “*Very Much*” (coded on a scale from 1 to 100). To create a single depression/anxiety scale, the four items for depression and the two items for anxiety were first combined within each subscale (mean of non-standardized items). The mean of these two subscales was then calculated for the depression/anxiety measure. This was done separately for each day. These specific symptoms were chosen because they focus strictly on the affective component of depression and anxiety, thus excluding social or somatic symptoms that may be correlated with depression or anxiety, and because they represent primary psychological components of PMDD in the DSM-5.

#### Time

Because the focus of the present study was on changes in symptoms across the menstrual cycle, the time and date of completion for each questionnaire was recoded to represent the proportion of each cycle that had passed since the first day of that cycle (day within cycle/total number of days in that cycle). Therefore, all participants, regardless of how many days their cycle lasted, were put on the same metric, ranging from 0 to 1 for each cycle (a 1 was then added to all days in the second cycle). Therefore, the time variable ranged from 0 to 2, with 0 corresponding to the first day of the first cycle, 1 corresponding to the last day of the first cycle, and 2 corresponding to the last day of the second cycle.

### Data Analyses

The main question addressed in this study is whether the magnitude and direction of menstrual cycle-related symptom change is associated with mean levels of those same affective symptoms. To address this question, two analytic approaches were used to define individual trajectory of affective symptoms across the two menstrual cycles. Both analytic approaches are based on the idea that the only meaningful patterns of change (besides no cyclical change) would be either a ∪ or an ∩ shaped trajectory, within each cycle, which corresponds to a W or an M shaped trajectory across the two cycles. It should be underlined that the estimation of these trajectories was done at the individual level, and then the individual-level regression slopes were saved and used for further analyses to create clusters and groups, as described below. All data analyses were conducted using JMP-Pro (27).

The first approach was a quartic polynomial regression in which the *Depression/Anxiety* score was regressed on the linear, quadratic, cubic, and quartic effects of time, thus yielding four regression coefficients that must be considered together for proper interpretation of the trajectory (these analyses were done using the “Fit Model” command in JMP, which simply specifies a general linear model). Quartic polynomials were used because we were testing for a repeating curvilinear effect across two cycles (i.e., W or an M shaped trajectory across the two cycles, as described above). To facilitate interpretation of these four coefficients a group-based approach was used in which the standardized regression coefficients (β's) for the linear, quadratic, cubic, and quartic effects, were saved separately for each individual (taken from separate individual regression analyses) and used as variables in a two-step cluster analysis. In the first step, to determine the optimal number of clusters, we examined the scree plots resulting from hierarchical cluster analyses (Ward's method), and examined the resulting clusters with the goal of identifying the fewest number of theoretically meaningful clusters. For example, based on the leveling-off of the scree plots, we could have included five or six clusters, although the additional clusters would have simply been a further division of participants showing different degrees of the W or M shaped trajectories across two cycles. In the second step, a *K*-means cluster analysis was used to classify each individual into a cluster for further analyses. *K*-means is a method of clustering observations (in our case participants with four observations representing the linear, quadratic, cubic, and quartic effects of time) by minimizing within-cluster variances (squared Euclidean distances) from a specific centroid (where number of centroids = k = number of clusters), and for which the number of clusters is already defined either empirically or by theory (determined to be *k* = 4 using Ward's method). Using these trajectory groups, mean-level group differences were then examined on the same symptoms. This approach was used previously by Kiesner [(9); those data will also be presented below as a point of reference], and the goal of the present study is to replicate and extend those earlier findings by using a second sample *and* a second analytic approach.

The second analytic approach was a cosine regression (6) in which the *Depression/Anxiety* score was regressed on the cosine function of the time variable: Cosine(2π·Time). Note that this specific cosine function constrains the shape of the trajectory to have either a ∪ or an ∩ shaped trajectory within each cycle, or a W or M shaped trajectory across the two cycles. This yields a single amplitude coefficient that provides information on both direction and magnitude of cyclical change. Numerically, the amplitude of the cosine wave is simply the difference between the average level across both cycles and the first peak/trough of the cosine wave function. Therefore, across two cycles, a cosine amplitude coefficient with a negative value corresponds to an M shaped wave (mid-cycle increase), a cosine amplitude coefficient with a positive value corresponds to a W shaped wave (perimenstrual increase), and a cosine amplitude coefficient equal to zero corresponds to no cyclical change. As with the polynomial regressions described above, these analyses were done using the “Fit Model” command in JMP, which simply specifies a general linear model. These analyses were conducted at the individual level and the regression coefficients were then saved and used for grouping participants, and for the regression analyses described below.

Because we wanted to compare the results from the cosine regressions and the polynomial regressions, we present analyses in which this continuous variable is used to create groups similar to those found in the polynomial regressions in terms of group size and direction of change. To create these groups, we used a series of cutoff scores to create 4 groups (within each sample) that were conceptually parallel, and similar in distribution, to the polynomial groups (e.g., to “mirror” those groups created with polynomial regressions). Thus, although the actual cutoff scores are somewhat arbitrary, the goal was to capture the same conceptual entities as those groups found in the polynomial analyses.

Although using cut-off scores may not be the optimal approach to handling a continuous variable, it does allow for a meaningful comparison with the other analytic approach. Moreover, further analyses will also treat the cosine coefficient as a continuous variable, testing for a curvilinear association between the individuals' cosine coefficient and their average level of negative mood. Thus, in the context of testing for an association between cyclical change and mean levels, cosine coefficients allow for both group-based analyses (using cutoff scores) and analyses treating cyclical change as a continuous variable, whereas polynomial regressions allow only for group-based analyses.

It should be noted that for both sets of analyses the intercept of the predictor variable (Time) was not centered. This was important in the polynomial regressions in order to have coefficients that could be meaningfully interpreted, and used in the cluster analyses, to identify trajectory groups. For example, analyses centering the time variable would not have provided interpretable patterns of coefficients associated with the W and M shaped trajectories (e.g., - + - +, and + - + -, respectively). It should be noted that centering or not centering results in equivalent model fits, as well as predicted values.

Following the above analyses used to create trajectory groups, three important sets of analyses were then conducted. First, we conducted regression analyses at the level of each trajectory group to verify that the slopes (or cosine coefficients) for each group were in the expected direction and were significant (or non-significant) when expected. Second, we conducted correspondence analyses (χ^2^) to test whether assignment to a specific trajectory group was consistent across analytic methods (within sample). Third, we compared trajectory groups on their mean level of depression/anxiety, thus addressing the main research question of the current study. Finally, we *replicated* these mean-level analyses treating the cosine regression coefficient as a continuous variable.

Finally, follow-up analyses were conducted to test for possible confounds/biases that could be attributable to effects of menstrual cycle length and missing data.

## Results

### Creation and Distribution of Polynomial Trajectory Groups

The polynomial trajectory groups for sample 1 have been previously analyzed and presented (9), and are presented here only as a point of reference for comparison. In these previous analyses the following four groups were identified: PMS1 pattern (strong perimenstrual increase in symptoms with a mid-cycle low; 24% of sample); PMS2 pattern (weaker perimenstrual increase in symptoms with a mid-cycle low; 37% of sample); Mid-Cycle pattern (mid-cycle increase in symptoms with a perimenstrual low; 13% of sample); and a non-cyclic pattern (no systematic change in symptoms associated with the menstrual cycle; 26% of sample).

The polynomial trajectory groups for sample 2 have not previously been presented, and results from sample 2 were remarkably similar to those from sample 1. Specifically, the same statistical analyses (cluster analyses using standardized regression coefficients) identified four groups also in sample 2, which demonstrated the same patterns and approximate distribution as sample 1. Specifically, the distribution of participants across the four trajectory groups was the following: PMS1 pattern (25% of sample); PMS2 pattern (33% of sample); Mid-Cycle pattern (16% of sample); and non-cyclic pattern (26% of sample).

**Figure 1:**
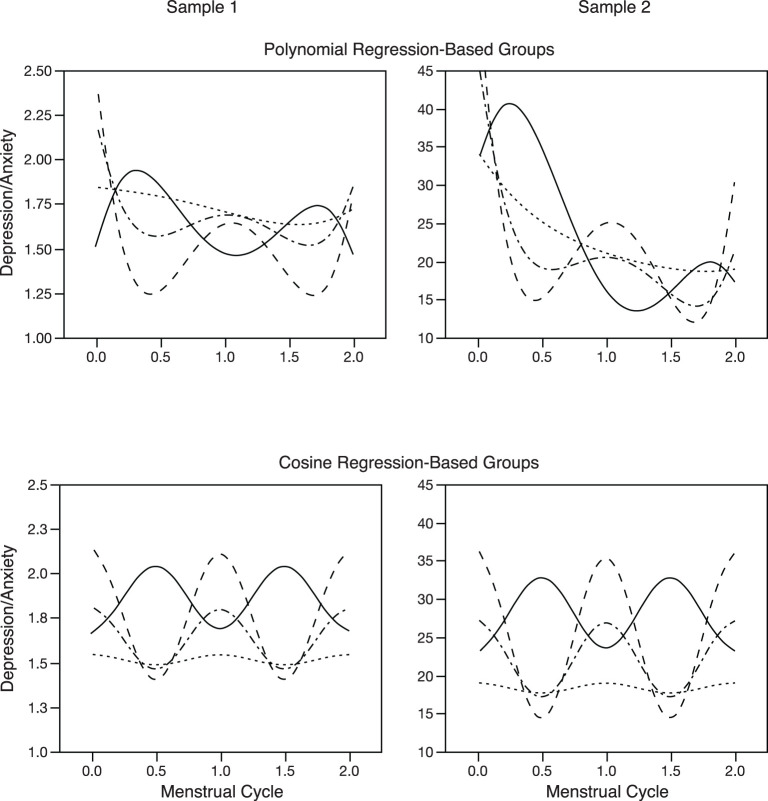
Trajectory of daily depression/anxiety scores across two menstrual cycles, plotted separately for the four trajectory groups, the two analytic techniques, and the two separate samples. “Depression/Anxiety” is the daily mean of the depression and anxiety scales, which are the means of individual questionnaire items. Top left plot showing polynomial regressions for sample 1, is used with permission (9).

Regression lines showing each group's trajectory of depression/anxiety across the two menstrual cycles, using polynomial regressions, are presented in the top half of Figure 1. It should be noted that although the four groups show similar trajectories across the two samples, sample 2 demonstrated an overall negative trend across the full 2 months, and across all groups, with higher levels of symptoms reported early in the study (early in cycle 1), regardless of trajectory group. Further analyses showed that even after partialling out the linear and quadratic effects of time across the full two-cycle time window prior to conducting the quartic polynomial regressions and cluster analyses (i.e., regressing *Depression/Anxiety* symptoms on both the linear and quadratic effects of time, across the entire two-cycle window, and then saving the residuals for the primary analyses), did not change the pattern of results regarding trajectory group analyses.

### Creation and Distribution of Cosine Trajectory Groups

**Figure 2:**
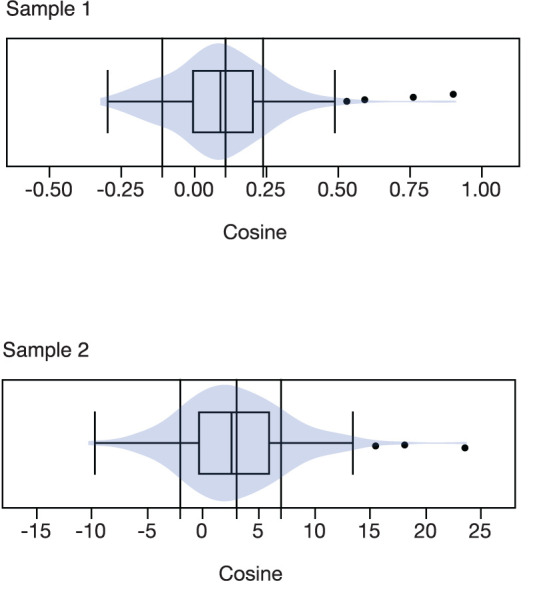
Box and contour plots, with group cut-off scores, for the cosine regression coefficients, presented separately for the two samples.

As described above, to create trajectory groups based on the cosine regression coefficients, cut-off scores were used. The distributions of the cosine regression coefficients and cutoff scores used to create groups (vertical lines) are presented in Figure 2.

For sample 1, the distribution of participants across the four groups was as follows: PMS1 pattern (17% of sample); PMS2 pattern (28% of sample); Mid-Cycle pattern (13% of sample); and non-cyclic pattern (43% of sample). For sample 2, the distribution of participants across the four groups was as follows: PMS1 pattern (18% of sample); PMS2 pattern (27% of sample); Mid-Cycle patter (12% of sample); and non-cyclic pattern (43% of sample).

Regression lines showing each group's trajectory of depression/anxiety across the two menstrual cycles, using cosine regressions, are presented in the bottom half of Figure 1. A comment should be made regarding the similarity in regression plots, across the two samples, for these cosine regressions. Specifically, apart from differences in scales, the shapes of cosine trajectories for the four groups look nearly identical across the two samples. This is an artifact of fitting a single and specifically shaped cosine function, which is mathematically constrained to vary only in direction and magnitude, and thus maintains the same basic shape across groups and samples. The polynomial regressions, on the other hand, allow a high degree of variability in overall shape, thus resulting in different shapes of the cyclical change across groups and samples.

### Verification of Expected Trajectories for Each Group

Analyses were conducted to verify that each group demonstrated significant slopes (or cosines) in the expected directions, or no significant slopes in the case of the non-cyclic group. Each analysis was conducted separately for each group and included participant as a random factor. Results for the polynomial analyses from sample 1 have previously been presented and are included here only for comparison. To simplify the presentation, a reduced set of results are presented (Table 2) and include only *t*-tests which provide the direction and significance of the relevant slopes (or cosine coefficients). Moreover, presenting the *t*-tests allows a reasonable comparison of effect size across samples and types of analyses. As is presented in Table 2, of the 40 effects tested, all effects that were expected to be significant were significant and in the expected direction.

**Table 2:** Regression coefficients indicating change in depression/anxiety across time, presented for each trajectory group separately, using polynomial and cosine regression analyses, including participant as a random factor.

		**Polynomial**				**Cosine**
		**Time**	**Time^**2**^**	**Time^**3**^**	**Time^**4**^**
		***t***	***t***	***t***	***t***	***t***
Sample 1	PMS1	−22.44***	22.00***	−21.35***	20.65***	18.35***
	PMS2	−12.51***	11.67***	−11.22***	10.92***	12.24***
	Non-cyclic	−0.23	0.02	−0.23	0.46	2.29
	Mid-cycle	7.26***	−8.53***	8.70***	−8.52***	−7.73***
Sample 2	PMS1	−22.50***	21.55***	−20.82***	20.16***	16.56***
	PMS2	−14.41***	12.04***	−10.93***	10.23***	11.00***
	Non-cyclic	−2.34	0.95	−0.59	0.47	2.06
	Mid-cycle	5.93***	−8.11***	8.08***	−7.52***	−6.00***

***p < 0.0001; given the high number of statistical comparisons in this table only effects with p < 0.0001 are considered significant. Time = linear effect of time, Time^2^ = quadratic effect of time, Time^3^ = cubic effect of time, Time^4^ = quartic effect of time.

### Correspondence Analysis Across Analytic Approach

Cross-tab χ^2^ analyses were conducted to test whether assignment to specific trajectory groups was consistent across analytic method (Table 3). For both samples a significant association was found between assignment based on the two grouping methods: χ^2^ = 158.34, *p* < 0.0001, for sample 1; and χ^2^ = 116.05, *p* < 0.0001 for sample 2. The high level of congruence is demonstrated by the percentage of participants that were assigned to the same groups using the different methods (52% for sample 1, and 56% for sample 2), with non-correspondence occurring in groups conceptually most similar to each other (e.g., PMS1 and PMS2; PMS2 and non-cyclic).

**Table 3:** Cross tabs of depression/anxiety trajectory groups estimated using polynomial regressions and cosine regressions, presented separately for two samples.

	**Sample 1**				**Sample 2**			
	**Cosine group**				**Cosine group**			
**Polynomial group**	**PMS 1**	**PMS 2**	**Non-cyclic**	**Mid-cycle**	**PMS 1**	**PMS 2**	**Non-cyclic**	**Mid-cycle**
PMS 1	25	20	6	0	22	11	8	0
PMS 2	11	32	36	0	8	26	20	0
Non-cyclic	0	7	38	11	0	6	30	6
Mid-cycle	0	0	11	16	0	1	12	13

*Sample 1: χ^2^ = 158.34, p < 0.0001; Sample 2: χ^2^ = 116.05, p < 0.0001*.

### Mean-Level Group Differences

Mean-level differences were tested by calculating the average level of depression/anxiety across both cycles for each participant (mean of all daily symptom reports across both menstrual cycles within each individual), then conducting a series of one-way ANOVAs using trajectory group as the only between-subjects factor. As previously reported (9), the trajectory groups based on polynomial regressions for sample 1 resulted in a significant effect of group (*F*_(3, 209)_ = 3.55; *p* < 0.05), and *post-hoc* analyses (Tukey) showed that the PMS1 group and the non-cyclic group were significantly different from each other, with the non-cyclic group showing the highest level of depression/anxiety, and the PMS1 group showing the lowest level (see Table 4). The parallel analyses for sample 2, however, yielded no significant effect of trajectory group (*F*_(3, 159)_ = 0.64; *p* < 0.60; Table 4), thus failing to replicate the previous finding in a second sample, when using polynomial regressions.

**Table 4:** Mean levels/intercepts of depression/anxiety (SDs in parentheses), for diverse trajectory groups based on polynomial and cosine regressions, presented separately for two samples.

	**Polynomial groups**	**Cosine GROUPS**
**Sample 1**		
PMS 1	1.48 (0.32)^A^	1.76 (0.30)^A^
PMS 2	1.65 (0.41)^AB^	1.63 (0.40)^AB^
Non-cyclic	1.72 (0.41)^B^	1.51 (0.37)^B^
Mid-cycle	1.67 (0.43)^AB^	1.86 (0.48)^A^
	*F*_(3, 209)_ = 3.55, *p* <0.05	*F*_(3, 209)_ = 7.48, *p* <0.0001
**Sample 2**		
PMS 1	20.98 (10.63)	24.88 (9.34)^A^
PMS 2	20.50 (10.84)	22.02 (11.69)^AB^
Non-cyclic	22.67 (12.34)	18.33 (10.86)^B^
Mid-cycle	23.85 (13.41)	28.15 (13.58)^A^
	*F*_(3, 159)_ = 0.64, *p* <0.60	*F*_(3, 159)_ = 5.05, *p* <0.002

*Groups with different letters differ significantly at p < 0.05, using Tukey post-hocs*.

Analyses comparing trajectory groups based on cosine analyses, on the other hand, yielded significant and very similar results for both sample 1 (*F*_(3, 209)_ = 7.48; *p* < 0.0001), and sample 2 (*F*_(3, 159)_ = 5.05; *p* < 0.002); and *post-hoc* analyses (Tukey) demonstrated an identical pattern of group differences across the two samples. Specifically, the PMS1 and Mid-Cycle groups showed the highest levels of depression/anxiety, and the non-cyclic group showed the lowest levels (significantly lower than both the PMS1 and mid-cycle groups), with the PMS2 group falling in the middle (see Table 4). Thus, in these analyses, participants at the extremes of the cosine coefficient distributions (PMS1 and Mid-Cycle) demonstrated the highest average levels (across two cycles) of depression/anxiety.

### Replication Treating the Cosine Coefficient as a Continuous Variable

**Figure 3:**
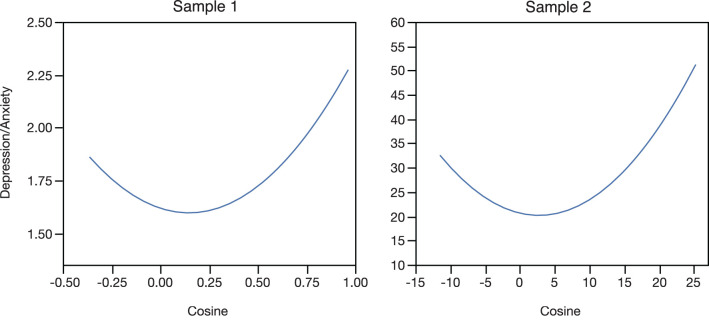
Polynomial regression lines of the individuals' average level of depression/anxiety regressed on their individual cosine coefficient, presented separately for the two samples. “Depression/Anxiety” is the individual-level mean across two menstrual cycles on the Depression/Anxiety scale, which is the mean of the depression and anxiety scales, which are the means of individual questionnaire items.

Because the cosine coefficient is more appropriately treated as a continuous variable, rather than a categorical variable, further analyses were conducted testing for a curvilinear effect of the cosine coefficient on level of depression/anxiety. For these analyses, mean level of *Depression/Anxiety* was regressed on the linear and quadratic effects of the cosine coefficient, expecting a curvilinear ∪-shaped function to be found, which would suggest that higher mean levels of depression are associated with higher degrees of cyclical change, regardless of whether the cyclical increases are observed perimenstrually or mid-cycle. Results were consistent with this hypothesis and very similar across samples. For sample 1, the overall model was marginally significant (*F*_(2, 210)_ = 2.59; *p* = 0.08), with a negative but non-significant linear effect (*t* = −1.46; *ns*), and a significant accelerating quadratic effect, (*t* = 2.28; *p* < 0.05); and for sample 2, the overall model was significant (*F*_(2, 160)_ = 5.34; *p* = 0.006), with a negative but non-significant linear effect (*t* = −1.32; *ns*), and a significant accelerating quadratic effect, (*t* = 3.03; *p* = 0.003). These curvilinear slopes are presented in Figure 3 and show that the extremes of the cosine distribution (mid-cycle and perimenstrual increases in symptoms) are associated with high and increasing mean levels of depression/anxiety, whereas the absence of cyclical change is associated with the lowest average level of symptoms. Note that the non-significant linear component of this slope is the estimated linear effect when the cosine coefficient is equal to zero, and thus does not represent the far left of the curvilinear slope, but the slope just above the value zero for the cosine coefficient.

### Does Cycle Length Predict Trajectory?

Because cycle length could be associated with symptom trajectory, two sets of additional analyses were conducted to test for this potential confound, and there was no consistent evidence that trajectory or trajectory group is associated with cycle length.

### Missing Data

Because some participants completed fewer daily reports than expected (the length of their two menstrual cycles was greater than number of daily reports), we tested whether the number of missing reports within each participant was associated with level of Depression/Anxiety (correlations) and the two sets of trajectory groups (ANOVAs), for each sample. None of these associations were significant (all *p*s > 0.23). Thus, the number of missing daily reports for each participant was unrelated to their level of Depression/Anxiety, or their assigned trajectory groups.

## Discussion

Using two longitudinal data sets, the present report describes the first evidence for a replicable association between menstrual cycle-related changes in affective symptoms and overall levels of affective symptoms, regardless of whether they occur perimenstrually or mid-cycle, thus highlighting the relevance of affective cyclicity in general work on affective disorders. Specifically, results from cosine analyses provide strong evidence that experiencing menstrual cycle-related changes in depression/anxiety is associated with higher mean levels of those symptoms, regardless of whether those cyclical increases occur perimenstrually or in the mid-cycle. Conversely, experiencing no significant cyclical change is associated with the lowest average levels of depression/anxiety. Thus, these findings appear to rule out the idea that menstrual cycle-related changes in affective symptoms are associated with lower levels of those symptoms, as had been suggested in an earlier publication (9). That is to say, it does not appear that the emotional highs outweigh, or even compensate for, the emotional lows associated with menstrual cycle-related changes in mood.

It is important to acknowledge the lack of replication across analytic approaches in the results regarding mean-level differences. However, because the cosine analyses appear to provide more consistent results across samples, and because they offer a more direct way of modeling cyclical change across time while providing a distribution of coefficient scores that can be easily studied in relation to other variables, this approach appears to provide significant advantages as compared to the polynomial regressions. These advantages are non-trivial in the context of studying cyclical change across many variables that are differentially present across different people and may causally influence each other.

An important insight provided by the cosine analyses regards the finding that both the PMS and the mid-cycle patterns of change in depression/anxiety are associated with higher mean levels of those symptoms. This suggests that the mid-cycle group-in addition to the already recognized PMS/PMDD pattern of cyclical change-should be recognized as a target for research that is associated with increased risk for chronic negative affective symptoms. That said, examination of the distribution of trajectory groups, and the distribution of the cosine coefficients, shows that the mid-cycle pattern is much less common than the PMS pattern, thus affecting fewer individuals. Nonetheless, considered along with previous studies (6, 9, 25, 26), the present report suggests that cyclical change in affective symptoms (PMS or mid-cycle patterns) should be considered as a risk factor for increased average levels of these same symptoms.

It should be noted that the present study is primarily relevant for identifying potential risk for broader and more stable changes in affective symptoms that are associated with cyclical changes in those symptoms. Thus, we do not suggest that the groups or prevalence rates be considered as indicative of clinical diagnoses or clinical prevalence rates. It should also be noted however, that although none of the participants had received a diagnosis of PMDD, an examination of individual data suggests that some clearly could have been, and that the lack of diagnoses is likely to be the result of a lack of training and information regarding PMDD among healthcare professionals in Italy. Moreover, it should be recognized that individual differences in mean levels and magnitude of cyclical change are *hidden* in the grouped data and by using regression slopes, which attenuate the peaks and troughs. Thus, the group-level regression slopes should not be considered as adequate representation of individual-level cyclical change.

Comparison with research by Pincus et al. (11) should also be made. As described in the introduction, those authors found no *significant* difference in average levels of negative mood across groups of females with brief recurrent depression, PMDD, and controls, although the controls showed the highest mean values of negative mood. Although the non-significance of their findings could be related to a small sample size, their control group did show the highest average level of negative mood and is thus in the opposite direction from our findings when using the cosine regressions. A possible explanation regards the inclusion of two clinical samples and a group of “normal controls” in the Pincus et al. study, as compared to the present study that was based on a community sample. However, this explanation is speculative, and based on the theories presented in the present paper we would still expect the PMDD patients in the Pincus et al. study to experience higher average levels of negative mood symptoms as compared to controls. Thus, further testing and replication will be necessary to clarify these differences.

A distinction should be made regarding increased risk for higher average levels of negative mood as found in the present study, and premenstrual exacerbation (PME) of other disorders. The present study specifically examined the association between an individual's average level of negative mood (across time) and the pattern of cyclical change they experience in those same mood symptoms across the menstrual cycle, whereas PME refers to a clinically significant premenstrual increase in symptoms of a chronic underlying disorder, such as borderline personality disorder [e.g., (28)]. Thus, although both concepts link menstrual cycle mood changes with chronic negative symptoms/outcomes, they are fundamentally different concepts. That said, one could hypothesize that at some point these concepts converge, for example, if the cyclical recurrence of symptoms raises the average level of symptoms to the point of experiencing a chronic disorder, which could then be further exacerbated by the continued recurrence of cyclical symptom change (i.e., PME). In a developmental psychopathology framework these effects would be defined as *cumulative consequences* and *contemporary consequences*, respectively (29).

Although the present study provides important insights regarding possible risk associated with affective change across the menstrual cycle, the sample was comprised only of young adult females in their twenties. Thus, the present study provides no information regarding adolescents or adults in their thirties or forties. Because different ages are associated with different degrees of experience with menstruation (i.e., adolescents have little experience whereas females in their forties have a great deal of experience) these other age groups could potentially demonstrate different patterns of results. For example, it could be hypothesized that, as females get older, the negative effects of cyclical change are further exacerbated, thus amplifying the effects as they age. Alternatively, adolescence may represent a sensitive period of development with regards to menstrual cycle-related symptoms and their sequalae, as described by Kiesner (12).

Additionally, because participants were recruited from the community rather than a clinic, it is possible that results do not generalize to a clinical population. However, it should be noted that recent work by Eisenlohr-Moul et al. (30) has shown that even among females with prospectively diagnosed DSM-5 PMDD, there are subtypes that differ in their temporal onset and resolution of symptoms within the menstrual cycle. Thus, although the questions and findings of the present study are very different from those presented (30), results do converge to suggest that there is significantly more heterogeneity among both clinical and non-clinical populations regarding temporal patterns of menstrual cycle-related symptoms. Moreover, research on depression and anxiety symptoms generally—and PMS/PMDD specifically—suggests that symptoms are most accurately conceptualized as dimensional risk processes rather than categorical states (31, 32). Nonetheless, future research should test for replication of the present findings across broader ranges of age and symptom severity.

A final limitation is that our analyses focused exclusively on *depression* and *anxiety*, thus excluding *irritability* and *mood swings*, which are also considered core emotional symptoms of PMDD. This exclusion, as well as the exclusion of other PMDD symptoms, limits the relevance of this study to PMDD as a disorder. That said, neither this limitation nor those described earlier present threats to the internal validity of the study.

In conclusion, the findings of this study provide important insights regarding potential risks associated with recurrent menstrual cycle-related increases in negative affect. These insights may help explain higher rates of major depression experienced by females than men (33–35), and may provide an easily identifiable causal factor that could be targeted in prevention programs. Moreover, considered in the context of past theories, such as the Kindling hypothesis (13, 14), allostatic load (15, 16), and the MCR-DAR model (12), menstrual cycle-related symptoms may create risk for long-term outcomes beyond the immediate effects of the menstrual cycle. Future research is clearly needed to examine these specific hypotheses which are of central importance because of their potential to provide a deeper understanding of depression and anxiety among females in general, and because of their potential to provide insights regarding focused prevention strategies that could benefit those who experience menstrual cycle-related symptoms at some point in their life.

## Data Availability Statement

The raw data supporting the conclusions of this article will be made available by the authors, without undue reservation.

## Ethics Statement

The studies involving human participants were reviewed and approved by Comitato Etico Della Ricerca Psicologica, University of Padova. The participants provided their written informed consent to participate in this study.

## Author Contributions

JK conceptualized and planned both studies. JK and TE-M conceptualized the research questions and co-wrote the manuscript. All authors contributed to data analyses and interpretation and approved the final version of the paper for submission.

## Funding

TE-M's contribution to this paper was partially supported by grants from the National Institute of Mental Health (K99109667).

## Conflict of Interest

The authors declare that the research was conducted in the absence of any commercial or financial relationships that could be construed as a potential conflict of interest.

## Publisher's Note

All claims expressed in this article are solely those of the authors and do not necessarily represent those of their affiliated organizations, or those of the publisher, the editors and the reviewers. Any product that may be evaluated in this article, or claim that may be made by its manufacturer, is not guaranteed or endorsed by the publisher.
